# A possible turning point for research governance in the life sciences

**DOI:** 10.1128/msphere.00407-25

**Published:** 2025-07-22

**Authors:** David R. Gillum

**Affiliations:** 1 University of Nevada Reno, Compliance and Research Administration 6851, Reno, Nevada, USA; University of Michigan, Ann Arbor, Michigan, USA

**Keywords:** biosafety, biosecurity, DURC, PEPP, gain-of-function, adaptive risk governance, virology, executive order, U.S. Policy

## Abstract

On 5 May 2025, the White House issued Executive Order (EO) 14292, halting federally funded “dangerous gain-of-function” research and rescinding the 2024 Dual Use Research of Concern (DURC) and Pathogens with Enhanced Pandemic Potential (PEPP) policy. While intended to strengthen biosafety and biosecurity, the EO introduces vague definitions, an abrupt 120-day policy development deadline, and politically charged rhetoric that could undermine trust and buy-in. Researchers, biosafety professionals, and institutions are left with a biosecurity policy vacuum after this EO, which is creating uncertainty across the scientific enterprise. This perspective considers the EO’s implications through empirical findings and practitioner insight and argues for a tiered, adaptive risk governance model grounded in scientific rigor, operational clarity, and institutional expertise to navigate future biosecurity challenges.

## PERSPECTIVE

On 5 May 2025, the White House issued Executive Order (EO) 14292, titled “Improving the Safety and Security of Biological Research,” framing it as a sweeping directive to strengthen biosafety and biosecurity oversight in the United States (1). The EO mandated an immediate pause on federally funded research labeled as “dangerous gain-of-function” (DGOF) that could result in “significant societal consequences,” rescinded the 2024 United States Government Policy for Oversight of Dual Use Research of Concern and Pathogens with Enhanced Pandemic Potential (2024 DURC/PEPP policy), and charged the Office of Science and Technology Policy (OSTP) with issuing a replacement policy within 120 days.

While the EO marks a decisive shift in Executive Branch oversight of high-risk biological research, it has also generated widespread confusion across the life sciences community. Issued just 1 day before the 2024 DURC/PEPP policy was set to take effect, the EO disrupted institutional efforts to implement that policy. Although the EO officially supersedes the 2024 framework, it does not explicitly repeal earlier guidance, such as the 2014 United States Government Policy for Institutional Oversight of Life Sciences Dual Use Research of Concern (2) or the 2017 Department of Health and Human Services (DHHS) Framework for Guiding Funding Decisions about Proposed Research Involving Enhanced Potential Pandemic Pathogens (P3CO) (3). This ambiguity has created uncertainty about which, if any, policies remain in force. This leaves federal agencies, research institutions, and investigators without clear guidance or guardrails for conducting life sciences research.

The 2024 DURC/PEPP policy represented a significant advancement over the 2014 and 2017 frameworks by integrating oversight of DURC and PEPP into a unified risk-tiered structure. It offered detailed implementation guidance (84 pages) and necessary definitions and recommended institutional procedures that enabled more consistent risk assessment. Even before the policy’s official issuance on 6 May 2024, institutions had begun to invest heavily in preparing for implementation. For example, many institutions took proactive steps beyond updates to paperwork. Several institutions conducted internal reviews of research inventories to determine applicability and categorization under the new policy and preemptively created detailed guidance documents and disseminated targeted updates to researchers.

In some cases, entire institutional protocols were rewritten to reflect biological agent tracking and containment requirements, even for research not currently subject to oversight. Institutions drafted contingency plans to treat unregulated research with high-risk pathogens as if subject to additional oversight. Some adapted or built infrastructure to accommodate the new expectations, such as converting lower-containment laboratories to high-containment facilities (e.g., biological safety level 3 or 4 containment) to meet anticipated needs. Others revised institutional biosafety committee (IBC) review processes to include more in-depth scrutiny at the proposal stage and required new or expanded research training on DURC and PEPP issues. These efforts represented a significant mobilization of institutional resources to meet the implementation deadline. EO 14292 abruptly halted these preparations, significantly disrupting institutional oversight plans.

IBCs, biosafety officers, virologists, microbiologists, and other researchers, as well as university administrators, now face a policy vacuum. Without clear guidance, laboratories working on influenza, antimicrobial resistance, synthetic biology, vaccine platforms, and other critical medical research are considering pausing or restructuring their programs. This operational paralysis threatens to delay pandemic preparedness, disrupt public health research, and produce unintended negative consequences for scientific progress and innovation.

However, EO 14292 also presents a significant inflection point that, if approached collaboratively with input from scientists, biosafety professionals, administrators, representatives supporting broader public engagement, and other key stakeholders, could spur a modernization of biosafety and biosecurity frameworks. By directing OSTP to revisit and rebuild the oversight framework for high-risk life sciences research, the EO creates a narrow policy window for modernizing U.S. biosafety and biosecurity. This moment demands more than a routine regulatory update: it calls for a fundamentally different paradigm in which biosecurity governance is handled in a transparent, scientifically grounded, and operationally feasible manner, and responsive to current and emerging biotechnological threats. If executed thoughtfully, the EO could usher in a more resilient, adaptive oversight model fit for the rapid advances of 21st-century biology.

The EO 14292 could be a pivotal point for meaningful reform. Yet, I argue that biosecurity governance must move away from rigid, top-down models and move to adaptive, risk-tiered systems that integrate institutional expertise, tacit knowledge of biosafety professionals and compliance personnel, along with scientists, and account for the dynamic nature of the U.S. research enterprise and bioeconomy. Such reform is possible and necessary to safeguard public health and scientific progress in an increasingly complex landscape.

## A LEGACY OF PATCHWORK GOVERNANCE

EO 14292 was not a surprise. U.S. biosafety and biosecurity governance has long been marked by fragmented oversight, inconsistent terminology, and regulatory frameworks that struggled to keep pace with scientific advancement. Over the years, policies such as the National Institutes of Health’s Guidelines for Research Involving Recombinant and Synthetic Nucleic Acid Molecules (4), federal select agent regulations (5), Occupational Safety and Health Administration’s Bloodborne Pathogens Standard (6), 2014 DURC policy, the 2017 P3CO framework, the 2024 DURC/PEPP policy, and others (7–9) have sought to address emerging risks. However, these efforts have been unevenly implemented between federally and privately funded research and between well-resourced and under-resourced institutions. These policies have also lacked integration across sectors, which has created a patchwork system that has left gaps in oversight and complicated compliance for researchers and institutions alike.

As a result, the life sciences community is now grappling with a maze of historical governance, conflicting definitions, and inconsistent institutional expectations. Empirical research that I have conducted, including surveys and interviews with biosafety officers and scientists, reveals significant variability in how institutions interpret and implement federal requirements, as well as in their capacity for compliance (10, 11). This fragmented governance structure has produced uneven oversight: in some cases, risk mitigation measures are overly restrictive, while in others, they fall short of addressing credible hazards. The outcome is a system that has struggled with coherence, predictability, and effectiveness, despite the substantial improvements introduced in the 2024 DURC/PEPP policy.

Previous efforts by the National Science Advisory Board for Biosecurity (NSABB) laid critical groundwork for defining and overseeing high-risk research. Their 2016 report “Recommendations for the Evaluation and Oversight of Proposed Gain-of-Function Research” (12) and the 2023 report “Proposed Biosecurity Oversight Framework for the Future of Science” (13) emphasized the need for scientifically grounded, regulatory-specific definitions. These reports advocated for categorizing research based on experimental outcomes, such as increased transmissibility, virulence, and host range in potential pandemic pathogens. Importantly, they recommended a tiered oversight framework grounded in transparent risk-benefit analysis and sustained stakeholder engagement. NSABB’s contributions helped define the contours of DURC and PEPP research and offered a robust foundation for OSTP to craft a modern, adaptive policy that avoids the definitional vagueness and implementation uncertainty now surrounding EO 14292. This current disruption, while destabilizing, has also removed institutional inertia and legacy constraints that previously made reform politically and administratively difficult. With prior policies seemingly rescinded and the OSTP tasked with crafting a new framework from scratch, there is an opportunity to consolidate authority, modernize definitions, and design a more coherent oversight system grounded in scientific evidence and operational realities.

## TRANSLATING POLICY INTO PRACTICE

For any biosecurity oversight framework to succeed, it must be designed with implementation at the institutional level in mind. Academic, clinical, community, government, and industrial laboratories differ widely in scope, resources, research focus, and risk tolerance. Institutional capacity to interpret and apply federal guidance often hinges not just on policy directives, but on institutional culture, staffing, and expertise. IBCs, biological safety professionals, environmental health and safety personnel, and responsible officials under the Federal Select Agent Program are essential to this system. These individuals are not passive observers; they are active participants in interpreting and operationalizing biosafety and biosecurity requirements. Their role in identifying and mitigating biological risk at the local level makes them indispensable partners in national biosecurity governance.

To be effective, EO 14292 and the framework that follows must do more than prescribe; they must empower. This means federal investment in scalable resources such as standardized training modules, shared incident reporting systems, biosafety and biosecurity tools and resources, and incentives for institutions to build and retain knowledgeable and experienced personnel. A one-size-fits-all approach will not work; oversight mechanisms must be flexible enough to accommodate institutional diversity, from research laboratories to production settings, while maintaining the highest standards for safety, security, and accountability.

In parallel, the success of any biosecurity policy also depends on public confidence. The coronavirus disease 2019 (COVID-19) pandemic elevated global awareness and scrutiny of high-risk life sciences research. In the wake of politicized debates over “gain-of-function” research, misinformation, distrust, and a lack of public understanding of biosafety and biosecurity protocols have emerged; trust must be rebuilt through transparency and meaningful engagement.

Future biosafety and biosecurity governance must clearly articulate why certain types of research are necessary, what risks they pose, and how they are mitigated. This includes greater transparency in funding decisions, public access to risk-benefit assessments, and clear visibility into how oversight decisions are made. Crucially, more open dialog is needed about the societal value of high-risk research and not just its dangers. Inclusive engagement is essential: scientists, ethicists, biosafety professionals, biosecurity experts, and representatives from affected communities must all sit at the table. Dismissing participants based on assumptions about their technical understanding or a blanket mistrust of scientists only deepens divides. Inclusive processes strengthen public oversight and enhance the legitimacy and durability of policy decisions (14, 15)

One persistent challenge in moving toward a unified oversight system is allocating technical expertise across agencies. The historical division of regulatory authority, such as the Centers for Disease Control and Prevention for human pathogens, the United States Department of Agriculture for plant and animal agents, and the National Institutes of Health for basic and applied research, reflects the prospect that no single agency holds comprehensive subject-matter expertise across all domains of the life sciences. However, this expertise problem is not insurmountable. With two other colleagues, I have recommended establishing a National Biosafety and Biosecurity Agency that would serve as a centralized, “one-stop shop” for biosafety and biosecurity regulation that could draw on interagency expertise through permanent advisory councils and rotating expert panels (16). This model would preserve necessary specialization while improving consistency, transparency, and coordination across sectors. IBCs, which already bridge diverse risk domains at the local level, could serve as implementation partners to help translate national policy into practice.

This approach would also align with the 2025 National Security Commission on Emerging Biotechnology recommendations, which recognizes the need for a whole-of-government strategy to modernize biotechnology oversight (17). The commission calls for a centralized coordinating entity to ensure coherent governance across the federal landscape, alongside sustained investment in biosafety infrastructure, risk assessment capabilities, and cross-sectoral coordination. The report also highlights the need to empower scientific institutions and the research workforce while balancing innovation with responsible stewardship of emerging technologies.

Ultimately, implementing effective governance is not just a technical challenge but a sociopolitical one. EO 14292 provides an opportunity to build a more trusted, accountable, and adaptive system for overseeing high-stakes science.

## RECOMMENDATIONS FOR A FUTURE POLICY FRAMEWORK

To capitalize on the opportunity presented by EO 14292, future policy must do the following.

- Replace vague terminology with scientific, vetted, operationalized, outcome-based definitions.
- Adopt a tiered risk governance approach to distinguish between low-, moderate-, and high-risk research.
- Empower institutional oversight bodies like IBCs with clearer authority, better tools, and federal support.
- Foster stakeholder engagement by including biosafety practitioners, biosecurity experts, scientists, public health experts, and members of the public in policy making.
- Support policy implementation with funding for training, compliance infrastructure, and applied research into best practices in biosafety and biosecurity.
- Promote public transparency through the open communication of regulatory rationale, decisions, and regular updates.
- Establish a National Biosafety and Biosecurity Agency to serve as a centralized authority that coordinates interagency expertise, standardizes oversight practices, and supports consistent implementation of biosafety and biosecurity across the life sciences enterprise.

## CONCLUSION

EO 14292 has created a disruption but also an opportunity. In its urgency, the EO reflects a broader recognition that our current oversight system is ill-equipped to handle modern bioscience challenges. The next iteration of federal policy must embrace adaptive governance, support institutional capacity, and prioritize definitional clarity. By focusing on an integrated, evidence-based, and practitioner-informed approach, the United States will continue to lead the world in biosafety, biosecurity, and scientific innovation. The coming months will determine whether EO 14292 becomes a source of regulatory paralysis or a catalyst for meaningful reform. The choice lies in how the OSTP and the broader scientific community respond, hopefully with collaboration, humility, and a shared commitment to the public good.
